# Maternal heat abatement during gestation alters growth, immunity, thermotolerance, and hepatic gene expression in beef offspring during backgrounding

**DOI:** 10.3389/fvets.2026.1849652

**Published:** 2026-05-29

**Authors:** Matheus L. Ferreira, Isabelle P. Siqueira, Marcelo Vedovatto, Wellison J. S. Diniz, Barbara R. dos Reis, Giancarlo P. Silva, Ashley K. Edwards, A. Lee Faulk, Aline C. dos Santos, Juliana Ranches

**Affiliations:** 1 Hill Farm Research Station, Louisiana State University, Homer, LA, United States; 2 Dean Lee Research and Extension Center, Louisiana State University, Alexandria, LA, United States; 3 Department of Animal Sciences, Auburn University, Auburn, AL, United States; 4 White Sand Research Unit, Mississippi State University, Poplarville, MS, United States; 5 Eastern Oregon Agricultural Research Center, Oregon State University, Burns, OR, United States

**Keywords:** beef calves, fetal programming, heat stress, shade, weaning

## Abstract

**Introduction:**

The objective of this study was to evaluate the fetal programming effects of removing maternal heat abatement during late gestation on offspring growth, thermotolerance, physiological responses, and liver gene expression during the backgrounding phase.

**Methods:**

Fifty-six Angus-cross calves [32 steers and 24 heifers, 225 ± 5.3 kg body weight (BW), 9 ± 1.5 months of age] were enrolled at weaning in a 112-day backgrounding study. Calves were born from cows provided (SS, *n* = 28) or not provided access to shade (NS, *n* = 28) during gestation. After weaning (d 0), calves were managed as a single group on bermudagrass pastures rotated biweekly. BW and blood samples were collected on d 0, 1, 3, 7, 14, 28, 48, 56, 84, and 112. Intravaginal temperature (IT) was recorded in heifers from d 48 to 56 and d 77 to 84. Liver biopsies were collected on d 56 for transcriptomic analysis. Data were analyzed using the GLIMMIX procedure of SAS, with maternal pasture as the experimental unit. Differential gene expressions (DEGs) were assessed using DESeq2 following read mapping and quality control.

**Results:**

SS calves were heavier at weaning and on d 14, 48, and 112 (*p* ≤ 0.03), although overall average daily gain did not differ between treatments (*p* = 0.89). From d 77 to 84, SS heifers exhibited lower IT than NS heifers (*p* = 0.03). Blood metabolites were not different between treatments (*p* ≥ 0.19), though SS calves had greater serum urea N on d 0, 3, and 7 (*p* = 0.04). Nine genes were differentially expressed, with eight downregulated (*AOX4, USP25, CFHR5, FGG, MBL2, FGB, GBE1, FGA*) and one upregulated (*FBLN2*) in NS heifers. Gene set enrichment analysis (FDR ≤ 0.05) showed enriched pathways related to heat shock protein binding and depleted of metabolic pathways in NS vs. SS calves. Functional overrepresentation showed enrichment of processes related to vasoconstriction and protein activation cascades in the NS versus SS calves.

**Conclusion:**

Removing maternal heat abatement during late gestation impaired offspring growth and thermoregulation during postweaning, enhanced humoral immunity, and altered liver gene expression.

## 1 Introduction

Heat stress is one of the most significant environmental challenges affecting beef production industries in the United States (1). Lack of heat abatement during gestation can induce fetal programming in dairy (2, 3) and beef cattle (4), a process where maternal stress alters developmental trajectories of the fetus, leading to long-term consequences on offspring performance, immunity, and adaptability.

Previous studies have shown that dairy calves born from heat-stressed cows are lighter at birth (5) and at weaning (6). They also exhibit reduced organ development, lower survivability, and decreased milk production for up to three lactations compared to calves born from cooled cows during late gestation (7). Beef cattle research has shown that removing maternal access to shade reduces calf birth (4, 8) and weaning weight (4). Despite overall impaired productivity and immunity, evidence is inconclusive regarding whether calves born to heat-stressed dams exhibit physiological changes related to heat dissipation later in life. Results in dairy cattle are inconsistent (9, 10), and evidence evaluating long-term thermotolerance following *in utero* heat stress in beef cattle remains limited.

Understanding how offspring born to heat-stressed beef cows cope with subsequent challenges after weaning is essential for improving resilience and productivity in beef systems under changing climatic conditions. For fall-calving herds, weaning typically occurs during the summer months, a period characterized by high ambient temperatures and humidity. During this time, calves experience multiple stressors associated with weaning management, including separation, dietary transition, and transportation (11). These stressors can be further aggravated by extreme heat, potentially compromising growth, immune function, and overall health. We hypothesized that greater heat stress exposure during late gestation, resulting from the removal of maternal heat abatement, would impair offspring postnatal growth, and alter endocrine and physiological responses to postweaning stressors, including thermoregulatory and immune challenges encountered later in life. Therefore, the objectives of this study were to evaluate the programming effects of removing maternal heat abatement during late gestation on: (1) offspring growth and thermotolerance, and (2) immunity, physiological responses, and liver gene expression during the backgrounding phase.

## 2 Materials and methods

The experiment was conducted at the Hill Farm Research Station, Louisiana State University Agricultural Center, Homer, Louisiana (32°45′18″N, 93°04′25″W) from May 2025 to Oct 2025. All animal handling and procedures were approved by the Institutional Animal Care & Use Committee of Louisiana State University (protocol A2024-04).

### 2.1 Animals, experimental design and management

Fifty-six Angus-cross calves [225 ± 5.3 kg body weight (BW), 9 ± 1.5 months of age] were enrolled in the study at weaning for a 112-day background period during the summer (June to September). Calves were born from cows given (SS, *n* = 28; steers = 16 and heifers = 12) or not given access to shade (NS, *n* = 28; steers = 16 and heifers = 12) during gestation.

Briefly, 72 black-hided Angus-cross cows were allocated into 1 of 12 bermudagrass pastures ‘common and coastal’ (*Cynodon dactylon*; 3 to 5 ha pasture/5 to 7 cows per pasture) provided or not with artificial shade from approximately 150 ± 20 d prepartum and 30 ± 10 d post-partum. Artificial shade structures (4.5 × 6.5 m) with black polypropylene shade cloth (80% ultraviolet radiation block) provided on average 4.9 m^2^ of shade per animal/pasture. Average respiration rate and intravaginal temperature differed between cows with access to shade and those without, averaging 82 vs. 110 ± 5.7 breaths/min and 38.3 vs. 38.7 ± 0.07 °C, respectively (Supplementary file). During the hottest hours of the day (1,400 to 1,800 h), intravaginal temperature was also lower in shaded compared with non-shaded cows (39.0 vs. 39.9 ± 0.08 °C, Supplementary file). Mean, minimum and maximum temperature (°C) and relative humidity (%) during the study were 21.5, 15.2 and 36.2; and 81.5, 34.2 and 100, respectively. Thus, treatments evaluated herein were calves born to cows exposed to different levels of heat load by removing heat abatement during gestation (i.e., shade vs. no shade). Dams were either supplemented or not with chromium propionate, which was added to their free-choice mineral. In this companion study, chromium was treated as a covariate due to its lack of influence on calf post-weaning responses. After ~30 days postpartum, cow-calf pairs were managed as a single group until weaning (at 7.5 ± 1 months of age) and provided with bermudagrass hay *ad libitum* during winter. Out of 72 cows, 56 calves were selected for this study during the background phase. More details on management and data collection for both cows and their calves from gestation to weaning are described in Ferreira et al. (4).

Approximately 21 days before weaning, calves were vaccinated against infectious bovine rhinotracheitis (IBR), parainfluenza-3 (PI_3_) virus, bovine viral diarrhea virus type 1 and 2 (BVDV-1 and 2), and bovine respiratory syncytial virus (2 mL subcutaneous, Bovi Shield Gold One Shot; Zoetis Inc., New York, NY).

At weaning (d 0), calves were immediately transferred to a partially covered drylot pen as a single group. Calves remained in the drylot pen for 7 d to overcome the weaning stress with free-choice access to bermudagrass hay [9.2% crude protein (CP) and 56% total digestible nutrients (TDN)]. On d 7, calves were turned out to bermudagrass pastures as a single group and rotated biweekly between 1 of 3 pastures (approximately 10 ha each). Calves were group-fed with an energy-protein supplement at 1% of BW from d 7 to d 112 (Table 1). All calves had free access to abundant natural shade, water, and complete mineral mix (14.8% Ca, 7.5% P, 18.8% NaCl, 1% Mg, 1% K, 3,600 ppm Zn, 3,600 ppm Mn, 1,200 ppm Cu, 12 ppm Co, 60 ppm I, 27 ppm Se, 662 IU/g vitamin A, 66/g IU vitamin D and 0.66 IU/g vitamin E; Purina Wind & Rain Storm All-Season 7.5 Complete).

**Table 1:** Ingredients of supplement offered to calves during background phase.

Ingredients	% as-fed
Soyhull pellets	25.5
Corn Gluten Feed	23.5
Cracked Corn	15
Dried distillers’ grains	15
Peanut Hulls pellets	12
Cottonseed Hull	4
Fortipel Calf^a^	2
Liquid conditioner	1.5
Calcium carbonate	0.8
Rumensin 5^b^	0.4
Salt	0.3

a FortiPel Calf—LNC (Livestock Nutrition Center/Auto-Max); custom vitamin-mineral premix carrier.

b Rumensin^®^ 5—Elanco Animal Health; monensin sodium ionophore for feed efficiency and coccidiosis control. Calves were group-fed with an energy-protein supplement [18% CP] at 1 g/kg of BW from d 7 to d 112.

### 2.2 Environmental data

Pastures were sampled to determine herbage mass and forage nutritive value on d 7, 28, 56, 84, and 112 (Table 2). Hand-plucked samples were used to evaluate forage nutritive value. Herbage mass (HM; kg DM/ha) were calculated using the double sampling technique (12) and herbage allowance (kg DM/kg BW) was determined by dividing the total pasture BW by the herbage mass (13). Forage and hay samples collected for nutritive value were dried in a forced-air oven (55 °C) for 72 h, ground at 2-mm sieve in a Wiley mill (model 3, Arthur H. Thomas, Philadelphia, USA) and sent to a commercial laboratory (Dairy One Forage Laboratory, Ithaca, NY) for determination of neutral detergent fiber (NDF; Van Soest et al. (14) and CP (method 984.13) by wet chemistry (15). TDN concentrations were calculated as described by Weiss et al. (16), and net energy for maintenance (NEm) and gain (NEg) by the equations proposed by NASEM (17).

**Table 2:** Chemical composition, herbage mass and allowance of bermudagrass pastures, and supplement chemical composition offered to beef calves during background phase.

Items^a^	Supplement^b^	Days relative to weaning				
		7	28	56	84	112
CP, % of DM	17.2	11.4	11.2	9.9	10.2	11.7
NDF, % of DM	37	68.2	69.7	68	66.4	69.9
TDN, % of DM	75	57	58	58	59	58
NEm, Mcal/kg of DM	1.81	1.12	1.14	1.16	1.17	1.14
NEg, Mcal/kg of DM	1.18	0.57	0.58	0.6	0.61	0.58
Herbage mass, kg DM/ha	—	5,232	5,054	3,776	5,734	1989
Herbage allowance, kg DM/ kg BW	—	1.83	1.72	1.26	1.75	0.60

a CP, crude protein; NDF, neutral detergent fiber; TDN, total digestible nutrients; NEm, net energy for maintenance calculated as described by Weiss et al. (16); NEg, net energy for gain calculated using the equations proposed by the NASEM (17). Herbage mass (HM) was determined using the double sampling technique described by Gonzalez et al. (12). Herbage allowance (HA) was determined by dividing the total pasture body weight by the herbage mass (13).

b Ingredients of supplemental feed offered to calves during background phase are listed in Table 1.

Ambient temperature and relative humidity were measured using a U23-001A data logger (Onset Computer Corp., Bourne, MA, USA) every hour during the entire study. Temperature and humidity parameters were used to calculate the thermal-humidity index (THI) and estimate potential heat stress. THI data was calculated daily according to Mader et al. (18).


THI=(0.8×T)+(%RH÷100)×(T−14.4)+46.4

where T = ambient temperature, RH = relative humidity.

**Figure 1:**
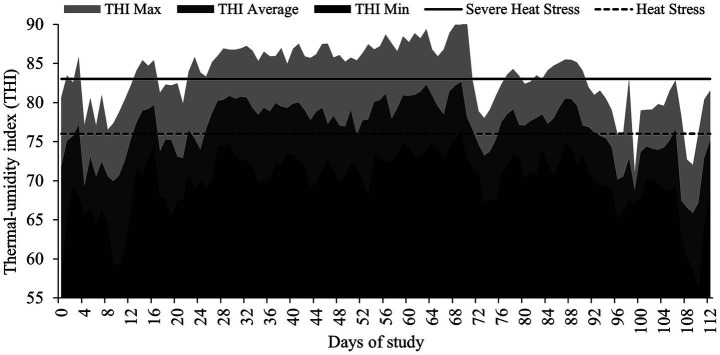
Maximum, average, and minimum daily THI from days 0 to 112 of the study. Calculated according to Mader et al. (18) as: THI = (0.8 × T) + (%RH ÷ 100) × (T – 14.4) + 46.4. Major heat stress, 79 ≤ THI < 84; and severe, THI ≥ 84, according to LCI (1970). Over the 112-day backgrounding phase, calves experienced moderate and major heat stress conditions for 70 and 35 days, respectively, based on average daily THI.

The THI values were interpreted according to the Livestock Weather Safety Index (LCI, 1970) for heat stress potential as: minimal, ≤ 74; moderate, 74 < THI < 79; major, 79 ≤ THI < 84; and critical, THI ≥ 84. Average, minimum, and maximum THI observed were averaged and reported daily (Figure 1).

### 2.3 Sample collections

Calf BW was recorded on d 0 (weaning), 3, 7, 14, 28, 48, 56, 84, and 112. Calf blood was collected on d 0, 1, 3, 7 14, 28, 56, 84, and 112 via jugular vein using commercial heparinized vacuum tubes for plasma harvest and tubes containing no additive for serum harvest (BD Vacutainer, 10 mL; Becton, Dickinson and Company, Franklin Lakes, NJ). Blood samples were placed on ice immediately after collection and centrifuged at 2,500 × g for 30 min at 4 °C to harvest serum and plasma. Serum and plasma samples were frozen at −20 °C and stored until further analyses. Serum samples were analyzed for concentrations of albumin, urea N, glucose, creatinine, alanine aminotransferase (ALT), aspartate aminotransferase (AST), gamma-glutamyl transferase (GGT). Plasma samples were analyzed for cortisol, insulin-like growth factor 1 (IGF-1), and haptoglobin.

Calf respiration rate was estimated biweekly in the pastures at 1,400 h from d 14 to d 112 by visually counting flank movements of each calf for 1 min (19). Intravaginal temperatures (IT) were collected from all heifers in the study (*n* = 12 per treatment) every 30 min for 7 consecutive days, from day 48 to day 56 and day 77 to day 84 using implantable loggers (iButton DS1921H-F5#; resolution of ± 0.5 °C; iButtonLink Technology, Whitewater, MI), as described by Burdick et al., (2012). Implantable loggers were placed intravaginally with a blank (hormone-free) controlled internal drug release device (Easy-Breed CIDR, Zoetis, Kalamazoo, MI, USA). Intravaginal temperature data collected on the days of sensor insertion and removal were excluded from the statistical analysis to avoid confounding effects associated with handling and movement to and from pastures. The remaining intravaginal temperature data were averaged in 30-min intervals, then averaged across d 49 to 55 and d 78 to 83 prior to statistical analysis.

Liver biopsies samples were collected on d 56 on calves randomly selected on day 0 [10 calves per treatment (5 steers and 5 heifers)] for transcriptomics analysis. After collection, the liver sample was stored in cryogenic tubes, snap-frozen in liquid nitrogen, and stored at −80 °C until RNA extraction.

### 2.4 Laboratory analysis

#### 2.4.1 Hormones and metabolites analyses

Serum albumin, total protein, urea N, glucose, and creatinine concentrations were determined using Beckman Coulter colorimetric and enzymatic colorimetric reaction kits. ALT, AST, and GGT activities were determined using Beckman Coulter enzymatic kits. All analyses mentioned above were determined using a Beckman Coulter AU480 Chemistry Analyzer (Beckman Coulter Inc., Brea, CA). Internal quality control and verification of performance within specific coefficient of variation for each assay were conducted daily. Globulins were determined by subtracting albumin from total protein. Serum urea N (SUN) was calculated as 46.67% of blood urea. Plasma cortisol and IGF-1 concentrations were measured using chemiluminescent enzyme immuno-assays (Immulite 2000; Siemens Medical Solutions Diagnostics, Los Angeles, CA) with intra- and inter-assay of 4.9 and 8.1%, and 3.1 and 7.6%, respectively.

Plasma haptoglobin was quantified in duplicate using a colorimetric assay based on the formation of the haptoglobin–hemoglobin complex, which is detected through changes in peroxidase activity (20). Arbitrary-unit values were converted into mg/mL according to Cooke and Arthington (21). Intra- and inter-assay coefficients of variation were 1.5 and 1.8%, respectively.

Antibody titers against IBR, PI3, and BVDV-1 and BVDV-2 viruses were determined on d 0, 14 and 56 in 12 calves per treatment (6 steers and 6 heifers) randomly selected on day 0. Analyses were conducted at the Oklahoma Animal Disease Diagnostic Laboratory (Stillwater, OK, USA) following procedures described by Rosenbaum et al. (22). Individual serum samples were evaluated for the greatest dilution that achieved complete cell protection against each virus, and results are reported as log₂ values. Serum neutralization titers <4 were considered negative and assigned a value of 0, whereas titers ≥4 were considered positive and assigned a value of 1. Seroconversion for each virus was calculated as the percentage of steers that exhibited a positive serum neutralization titer response (23).

#### 2.4.2 Liver transcriptome: RNA extraction, library preparation, sequencing, and raw data processing

Total RNA was extracted from the liver using Trizol (Qiagen Germantown, MA, USA), followed by DNase treatment, according to the manufacturer’s protocol. The purity and integrity of the RNA samples were assessed with the Agilent 2100 Bioanalyzer (Agilent, CA) and agarose gel electrophoresis. All samples met the quality thresholds for library preparation (RNA integrity number > 7.0).

Strand-specific RNA libraries were prepared using the NEBNext Ultra II Directional RNA Library Prep Kit for Illumina (New England BioLabs, Ipswich, MA, USA) with PolyA mRNA enrichment. Paired-end libraries were sequenced on the Illumina NovaSeq X Plus platform, with 150 bp reads and a 20 M reads/sample depth. Novogene Co., Ltd. (Nanjing, China) performed the library preparation and sequencing. Raw data quality control was performed using FastQC v. 0.11.9. The MultiQC v. 1.17 was used to aggregate the read statistics from FastQC. The reads were mapped to the ARS-UCD1.3. *Bos taurus* reference genome using the STAR aligner v.2.7.5. Mapped reads were counted using the quantMode GeneCounts flag from STAR. Post-mapping quality control was conducted with MultiQC and edgeR v. 4.0.3.

### 2.5 Statistical analysis

#### 2.5.1 Growth, thermotolerance and physiological responses variables

Data was analyzed using the GLIMMIX procedure of SAS (SAS Institute Inc., Cary, NC, USA, version 9.4) considering maternal pasture as experimental unit for all dependent variables. Pasture (treatment) was considered the random effect in all statistical analysis. Kenward-Roger method of approximation was used to adjust degrees of freedom for the test of fixed effects. Calf variables were analyzed for the fixed effects of maternal treatments (shade *vs*. no shade during gestation), day of sampling, treatment and day interaction. Sampling day was considered repeated measures over time for calf BW, RR, and blood variables, and sampling hour for IT. The best covariance structure was chosen based on the lowest Akaike Information Criterion specific for each variable (chosen from compound symmetry, heterogeneous compound symmetry, autoregressive, and heterogeneous autoregressive). Seroconversion status for antibody titers was modeled as binomial distribution using the GLIMMIX procedure, analyzed as a repeated measure and tested for the fixed effects of maternal treatment, day and treatment and day interaction. Additionally, chromium supplementation to damns during gestation period was included as a fixed effect but no significant differences that observed for all variables herein analyzed (*p* ≥ 0.18). To ensure further chromium supplementation was not confounded with maternal shade treatment, calves were equally and independently distributed across treatment groups. Calf sex and age were included as covariates for all variables. Least square means were separated using least significant difference. Differences were set at *p* ≤ 0.05 and tendencies were determined if *p* > 0.05 and ≤ 0.10.

#### 2.5.2 Liver transcriptomics: differential expression, functional over-representation and gene set enrichment analyses

Principal component analysis was conducted using the factoextra package (version 1.0.7) in R to identify potential batch effects. Differentially expressed genes (DEGs) were identified using DESeq2 v. 1.30.1, which applies a negative binomial distribution to model the RNA-Seq data. A pairwise comparison between the NS and SS groups was performed, adjusting for sex (~Sex + Treatment). Additionally, differential expression was assessed within each sex and for the treatment by sex interaction. Gene annotation was performed using the biomaRt package (version 2.58.2) based on the *Bos taurus* ARS-UCD1.3 genome (Ensembl release 113). Genes with FDR ≤ 0.1 were considered differentially expressed and were classified as up or downregulated based on the sign of the log2 fold change in the NS group.

Functional enrichment analyses were performed using two complementary approaches. First, over-representation analysis (ORA) was conducted using the list of differentially expressed genes (DEGs) to identify significantly enriched Gene Ontology terms and KEGG pathways. Second, gene set enrichment analysis (GSEA) was performed using the complete ranked gene list based, allowing detection of coordinated changes in predefined biological pathways without applying a differential expression cutoff. Functional enrichment analysis of DEGs was performed only for female specific DEG set (*n* = 9) using ShinyGO v0.85.1. to identify over-represented biological processes and KEGG pathways. The background gene set consisted of all expressed genes retained after QC filtering and FDR ≤ 0.05 was defined as significant. ShinyGO uses curated annotation databases and a hypergeometric test framework with false discovery rate (FDR) correction to assess gene ontology (GO) terms and pathway enrichment. Pathways and GO terms with FDR ≤ 0.05 were considered significantly enriched. Because only two DEGs were identified in the overall analysis, pathway enrichment was not performed for that comparison.

To further investigate the functional role of expressed genes, a Gene Set Enrichment Analysis (GSEA) was implemented through WebGestalt (WEB-based Gene SeT AnaLysis Toolkit). The GSEA allow to identify over-represented KEGG pathways and gene ontology terms (GO) underlying biological processes and molecular function at the top or bottom of the ranked list of genes. The GSEA method considered all genes tested for differential expression. This approach was used to identify group of genes (gene sets) acting on common pathways rather than DEGs individually (24). To generate the ranked list, genes were ordered using the equation: rank = [sign (log_2_FC) × − log_10_(*p* − value)], where the direction of change was determined by the sign of the fold change and the strength of evidence was reflected by the *p*-value. In the GSEA output, enrichment for each gene set was expressed as a normalized enrichment score (NES), which adjusts the enrichment score to account for differences in gene set size and other dataset-specific characteristics. A positive NES indicates that a gene set is concentrated toward the top of the ranked list (genes with positive fold changes), whereas a negative NES reflects enrichment among genes with negative fold changes (24).

## 3 Results

### 3.1 Thermal-humidity index

Mean, minimum, and maximum temperature (°C) and relative humidity (%) during the study were 27.2, 13.3, and 39.8; and 79.5, 37.8, and 100, respectively. Over the 112-day backgrounding phase, calves experienced moderate and major heat stress conditions for 70 and 35 days, respectively, based on average daily THI (Figure 1).

### 3.2 Calf growth and thermotolerance

**Figure 2:**
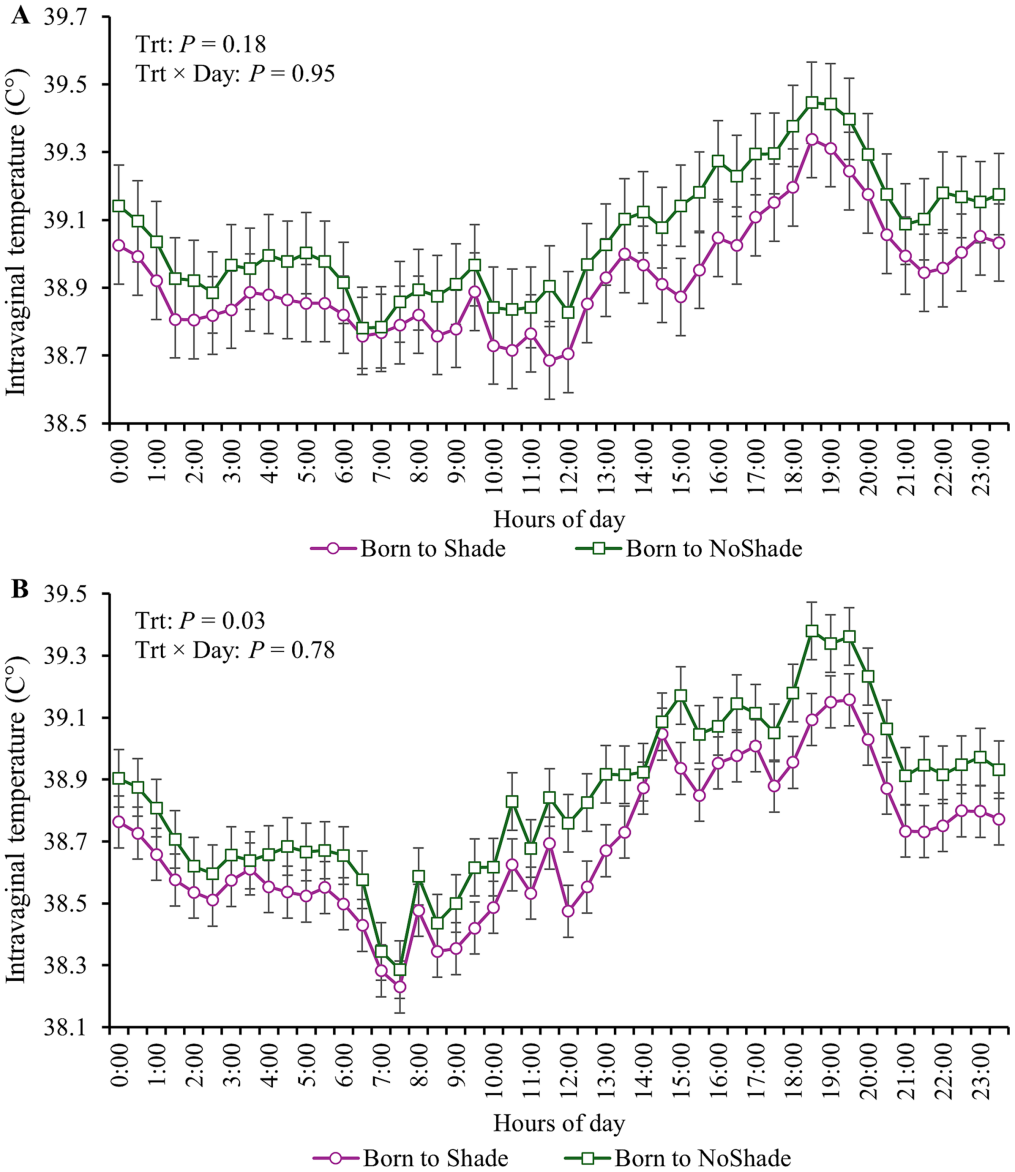
Intravaginal temperature (°C) from d 48 to 56 **(A)** and d 77 to 84 **(B)** heifers during background phase born to shade (*n* = 12) or no shaded cows (*n* = 12) during gestation under heat stress conditions. Intravaginal temperature was averaged at 30-min intervals and then across days 46 to 56 **(A)** and days 77 to 84 **(B)** before statistical analysis. SS heifers had a lower average IT compared to NS heifer calves (*p* = 0.03) from day 77 to day 84, but no differences were observed from day 48 to day 56 (*p* = 0.18).

Calves born to shaded cows were heavier at weaning, on d 14, 48 and 112 postweaning (*p* ≤ 0.03; Table 3). NS calves tended to lose more weight from weaning to d 3 postweaning (*p* = 0.09). No difference between treatments was detected for the overall average daily gain (ADG; *p* = 0.89). SS heifers had a lower average IT compared to NS heifer calves (38.6 vs. 38.9 ± 0.04 °C, *p* = 0.03, Figure 2A) from day 77 to day 84, but no differences were observed from day 48 to day 56 (*p* = 0.18; Figure 2B).

**Table 3:** Growth performance of background calves born to cows given or not access to shade during gestation under heat stress conditions.

Item	Maternal treatments		SEM	*p*-value
	Born to shade	Born to no shade		Treatment
Weaning BW	234	217	5.19	0.03
BW change d0 to d3	−2.8	−8.0	2.14	0.09
BW change d0 to d7	−11.8	−11.9	1.41	0.97
BW change d7 to d14	9.0	11.2	1.07	0.15
Day 14 BW	243	229	5.38	0.04
BW change d14 to d48	15.3	13.5	1.43	0.36
Day 48 BW	258	242	5.66	0.05
BW change d48 to d112	53.9	52.6	4.09	0.82
Final BW	287	269	7.34	0.03
Average daily gain, kg/d	0.50	0.45	0.05	0.89

Calves were born from cows given (*n* = 28; steers = 16 and heifers = 12) or not given access to shade (*n* = 28; steers = 16 and heifers = 12) during gestation. After weaning (d 0), calves were kept in a drylot pen for 7 d, then calves were divided into 1 of 4 bermudagrass pastures rotated biweekly. Calves were group-fed with an energy-protein supplement [18% crude protein (CP)] at 1 g/kg of BW from d 7 to d 112. Average daily gain calculated from d 0 to d 112.

### 3.3 Physiological responses and immunity

**Figure 3:**
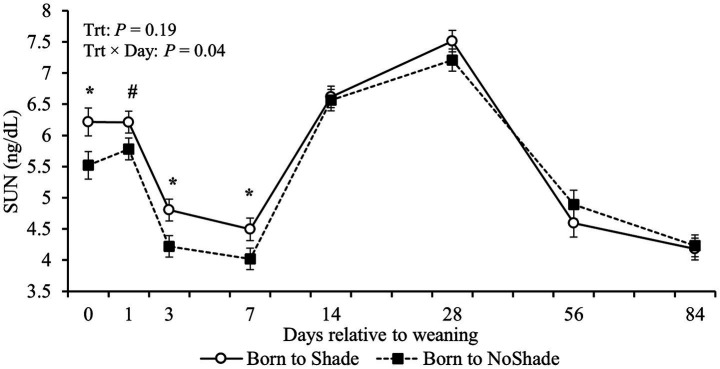
Serum urea nitrogen (SUN) concentration in background calves born to cows given (*n* = 28) or not given access to shade (*n* = 28) during gestation under heat stress. * Shows difference (*p* ≤ 0.05) and # shows tendency (0.10 ≥ *p* > 0.05) between treatments withing day. Effect of maternal treatment × day interaction was detected for SUN concentrations (*p* = 0.04), in which calves born to shaded cows had greater SUN concentrations on d 0, 3, and 7 (*p* ≤ 0.02) and tended to be greater on d 1 (*p* = 0.08) compared to calves born to not shaded cows.

No differences between maternal treatment (*p* ≥ 0.19) and maternal treatment × day (*p* ≥ 0.33) were detected for concentrations of glucose, total proteins, globulins, creatinine, and IGF-1 (Table 4). Effect of maternal treatment × day interaction was detected (*p* = 0.04) for SUN concentrations (Table 4), in which SS calves had greater SUN concentrations on d 0, 3, and 7 (*p* ≤ 0.02; Figure 3) and tended to be greater on d 1 (*p* = 0.08) compared to NS calves. No differences between maternal treatment (*p* ≥ 0.65) or maternal treatment × day (*p* ≥ 0.21) were detected for activity of enzymes AST, ALT, and GGT (Table 4).

**Table 4:** Blood metabolites, enzymes and hormones of background calves born to cows given or not access to shade during gestation under heat stress conditions.

Item	Maternal treatments		SEM	*p*-value	
	Born to shade	Born to no shade		Trt	Trt × Day
Glucose, mg/dL	53.3	53.8	1.468	0.81	0.71
Total proteins, g/dL	5.74	5.78	0.056	0.63	0.39
Albumin, g/dL	2.78	2.70	0.029	0.80	0.33
Globulins, g/dL	2.96	3.01	0.052	0.51	0.53
SUN, mg/dL	5.53	5.30	0.124	0.19	0.04
Creatinine, g/dL	1.21	1.19	0.027	0.57	0.44
AST, U/L	78.9	81.1	1.895	0.67	0.82
ALT, U/L	38.02	32.78	8.211	0.65	0.21
GGT, U/L	12.53	12.54	0.646	0.98	0.81
IGF-1, ng/mL	129.1	120.4	4.774	0.20	0.56

Calves were born from cows given (*n* = 28; steers = 16 and heifers = 12) or not given access to shade (*n* = 28; steers = 16 and heifers = 12) during gestation. After weaning (d 0), calves were kept in a drylot pen for 7 d, then calves were divided into 1 of 4 bermudagrass pastures rotated biweekly. Calves were group-fed with an energy-protein supplement [18% crude protein (CP)] at 1 g/kg of BW from d 7 to d 112.

No differences between maternal treatment (*p* ≥ 0.72) or maternal treatment × day (*p* ≥ 0.54) were detected for concentrations of cortisol and haptoglobin (Table 5). NS calves exhibited greater titer concentrations (*p* = 0.03) and seroconversion (*p* < 0.0001) for BVDV-1, compared to SS calves. No differences between maternal treatments (*p* ≥ 0.32) or interaction treatment × day (*p* ≥ 0.28) were observed for titer concentrations and seroconversion for IBR, PI3, BVDV-2, and BRSV (Table 5).

**Table 5:** Stress markers, serum titers concentrations and seroconversion of background calves born to cows given or not access to shade during gestation under heat stress conditions.

Item^a^	Maternal treatments		SEM	*p*-value	
	Born to shade	Born to no shade		Trt	Trt × day
Stress markers					
Cortisol, ng/mL	2.22	2.23	0.148	0.95	0.61
Haptoglobin, mg/dL	0.49	0.47	0.034	0.72	0.54
Titer concentration, log_2_					
IBR	2.16	2.21	0.084	0.69	0.85
PI_3_	2.40	2.41	0.138	0.97	0.28
BVDV-1	2.69	3.24	0.179	0.03	0.08
BVDV-2	3.74	3.92	0.258	0.55	0.45
BRSV	2.40	2.55	0.106	0.32	0.92
*S*eroconversion, %					
IBR	10	14	4.25	0.61	0.84
PI_3_	24	21	3.55	0.97	0.71
BVDV-1	31	48	5.59	<0.0001	0.88
BVDV-2	57	52	2.21	0.92	0.98
BRSV	31	33	4.94	0.61	0.84

a IBR, infectious bovine rhinotracheitis; BVDV-1, bovine viral diarrhea virus type 1; BVDV-2, bovine viral diarrhea virus type 2; BRSV, bovine respiratory syncytial virus. A vaccine against these viruses (2 mL subcutaneous; Bovi Shield Gold One Shot; Zoetis, Parsippany, NJ) was administered in all calves on d − 21. Calves were born from cows given (*n* = 28; steers = 16 and heifers = 12) or not given access to shade (*n* = 28; steers = 16 and heifers = 12) during gestation. Antibody titers against IBR, PI3, and BVDV-1 and BVDV-2 viruses were determined on d0, 14 and 56 in calves 12 calves per treatment (6 steers and 6 heifers) randomly selected on day 0. Cortisol and haptoglobin were measured on days d 0, 1, 3, 7 14, 28, 56.

### 3.4 Liver transcriptomics

#### 3.4.1 Differential expression and functional over-representation analyses

The RNA-Seq approach generated, on average, 26.437 million clean reads per sample (ranging from 21 to 32.3 M reads per sample). On average, 97% of the total reads were uniquely mapped to the reference genome. Following post-mapping quality control by removing non expressed or lowly expressed genes (minimum 10 cpm in 70% of samples), 14,680 out of 36,075 genes remained in the 20 samples for differential expression analysis. When adjusted for sex, only the genes *USP25* and *ENSBTAG00000062287* were downregulated in the NS group (FDR ≤ 0.1). When evaluating sex-specific effects, no DEGs were detected between treatments in males, and no significant treatment by sex interaction were observed. However, nine DEGs were identified in females; of those, eight were downregulated [*AOX4*, *USP25*, *CFHR5*, *FGG*, *MBL2*, *FGB*, *GBE1*, *FGA*] in the NS animals and one was upregulated [*FBLN2*].

**Figure 4:**
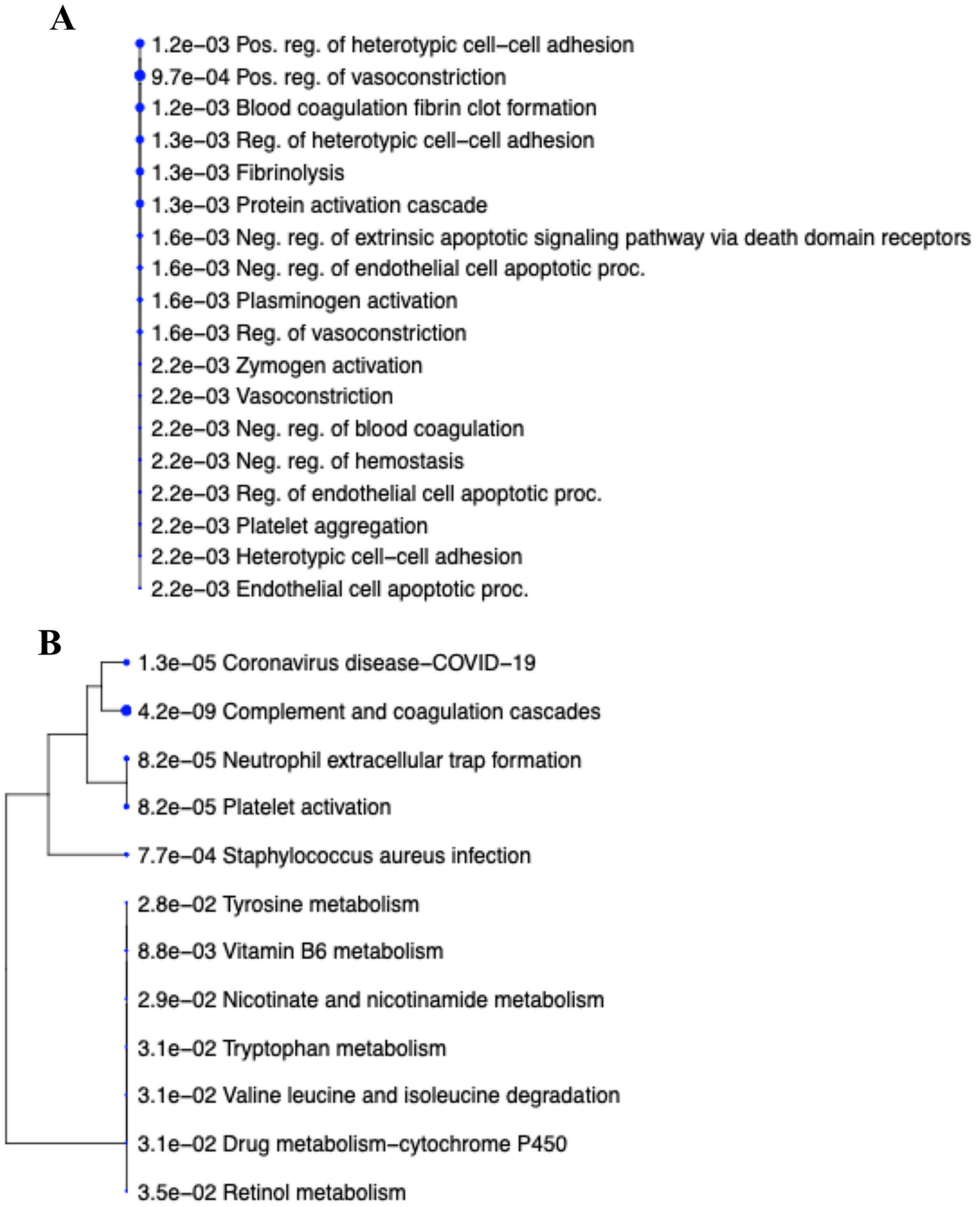
Functional over-representation analysis of differentially expressed genes in the liver tissue of beef heifers. **(A)** Biological processes and **(B)** KEGG pathways over-represented were based on the DEGs from NS heifers vs. SS heifers. Liver biopsies were performed on d 56 on calves randomly selected on day 0 for transcriptomics analysis [calves born from cows without access to shade (NS, *n* = 10; steers = 5 and heifers = 5) or given access to shade (SS, *n* = 10; steers = 5 and heifers = 5)]. The terms are hierarchically arranged based on functional similarity. The bigger the blue dot, the more significant the term is (FDR ≤ 0.05) based on the comparison of NS vs. SS calves.

Functional over-representation analysis of DEGs in liver tissue identified significant biological processes and pathways associated with the observed transcriptional changes using two complementary approaches. Over-representation analysis based on the DEG from the female specific DEG set (*n* = 9) identified biological processes related to vascular regulation, hemostasis, and cellular adhesion (FDR ≤ 0.05). Over-represented terms included regulation of vasoconstriction, blood coagulation and protein activation cascades (Figure 4). Notably, KEGG pathways also included vitamin metabolism (e.g., vitamin b6 metabolism and retinol metabolism) and immune processes (e.g., complement and coagulation cascades). Although the number of DEGs identified in females was limited, the enrichment analysis revealed convergent functional categories, supporting the interpretation that these genes are involved in related biological processes rather than representing random transcriptional variation. However, these findings should be interpreted cautiously and considered exploratory until validated in larger datasets or independent populations. Over-represented pathways annotated as viral infection or cancer-related likely reflect limitations in cross-species pathway annotation rather than true disease processes in cattle. Because KEGG pathway terms are derived from human datasets, they often group genes into disease-associated categories that also capture conserved biological functions such as immune signaling and inflammatory regulation. Therefore, emphasis should be placed on shared functional gene networks rather than disease-specific interpretations.

#### 3.4.2 Gene set enrichment analyses

**Figure 5:**
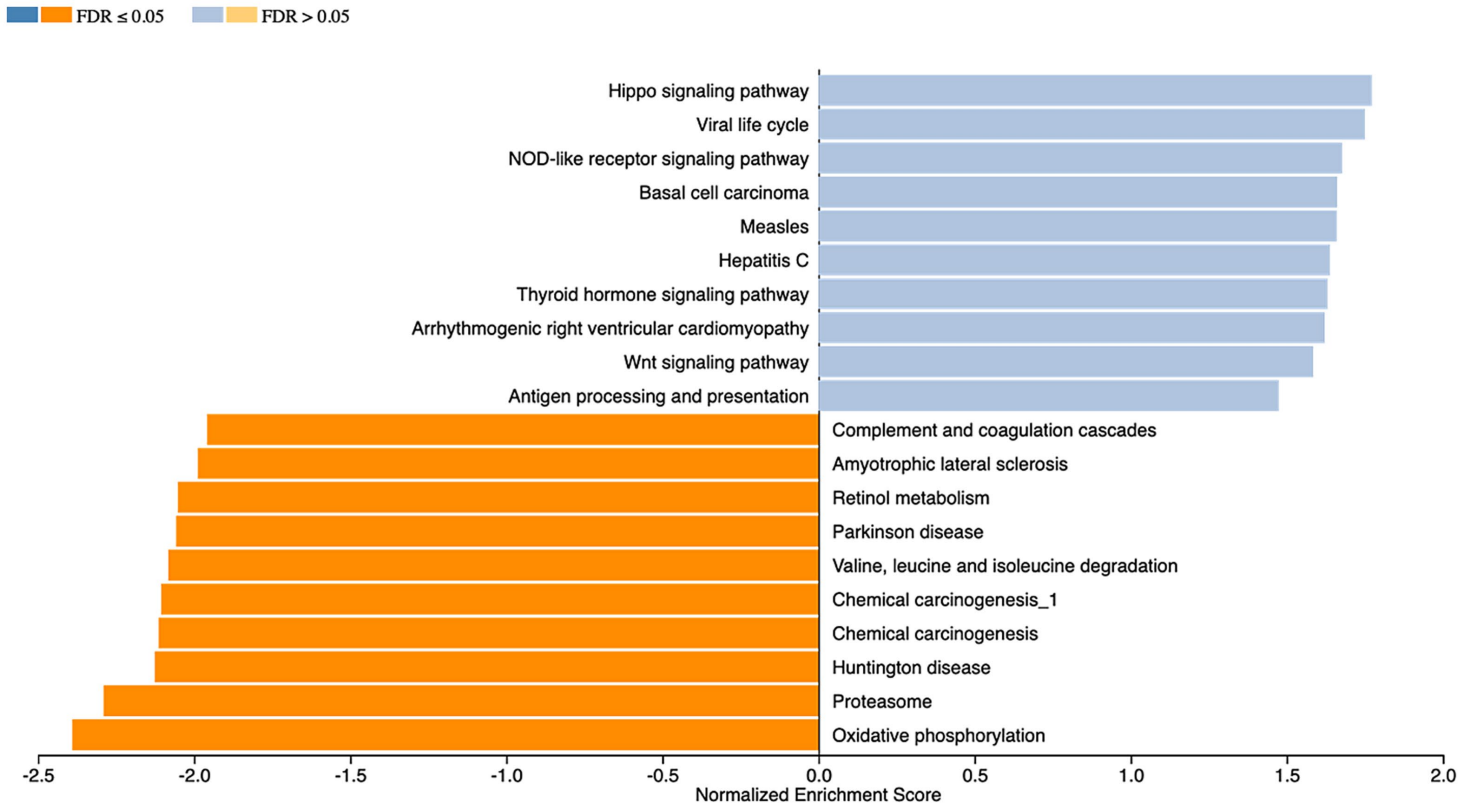
Gene set enrichment analysis-based pathway on KEGG terms of expressed genes in the liver of beef calves. Liver biopsies were performed on d 56 on calves randomly selected on day 0 for transcriptomics analysis [calves born from cows without access to shade (NS, *n* = 10; steers = 5 and heifers = 5) or given access to shade (SS, *n* = 10; steers = 5 and heifers = 5)]. The normalized enrichment score (NES) of top enriched (blue bars) and top depleted (orange bars) pathways are based on the comparison of NS vs. SS calves (FDR ≤ 0.05).

In addition to the over-representation analysis of DEGs, a gene set enrichment analysis (GSEA) of the complete ranked gene expression dataset identified additional pathways showing coordinated but modest expression shifts that were not captured at the individual gene level. KEGG pathway analysis based on the GSEA revealed distinct differences in hepatic gene expression between NS calves and SS calves (Figure 5). In NS calves, enriched pathways (positive NES) primarily involved immune and signaling functions, including thyroid hormone signaling, NOD-like receptor signaling pathway and antigen processing and presentation, but were not significant (FDR > 0.05). In contrast, depleted pathways (negative NES) in NS calves consisted largely of metabolic pathways, including oxidative phosphorylation, proteasome function, valine, leucine, and isoleucine degradation, retinol metabolism, and pathways associated with mitochondrial or protein-degradation processes (FDR ≤ 0.05).

**Figure 6:**
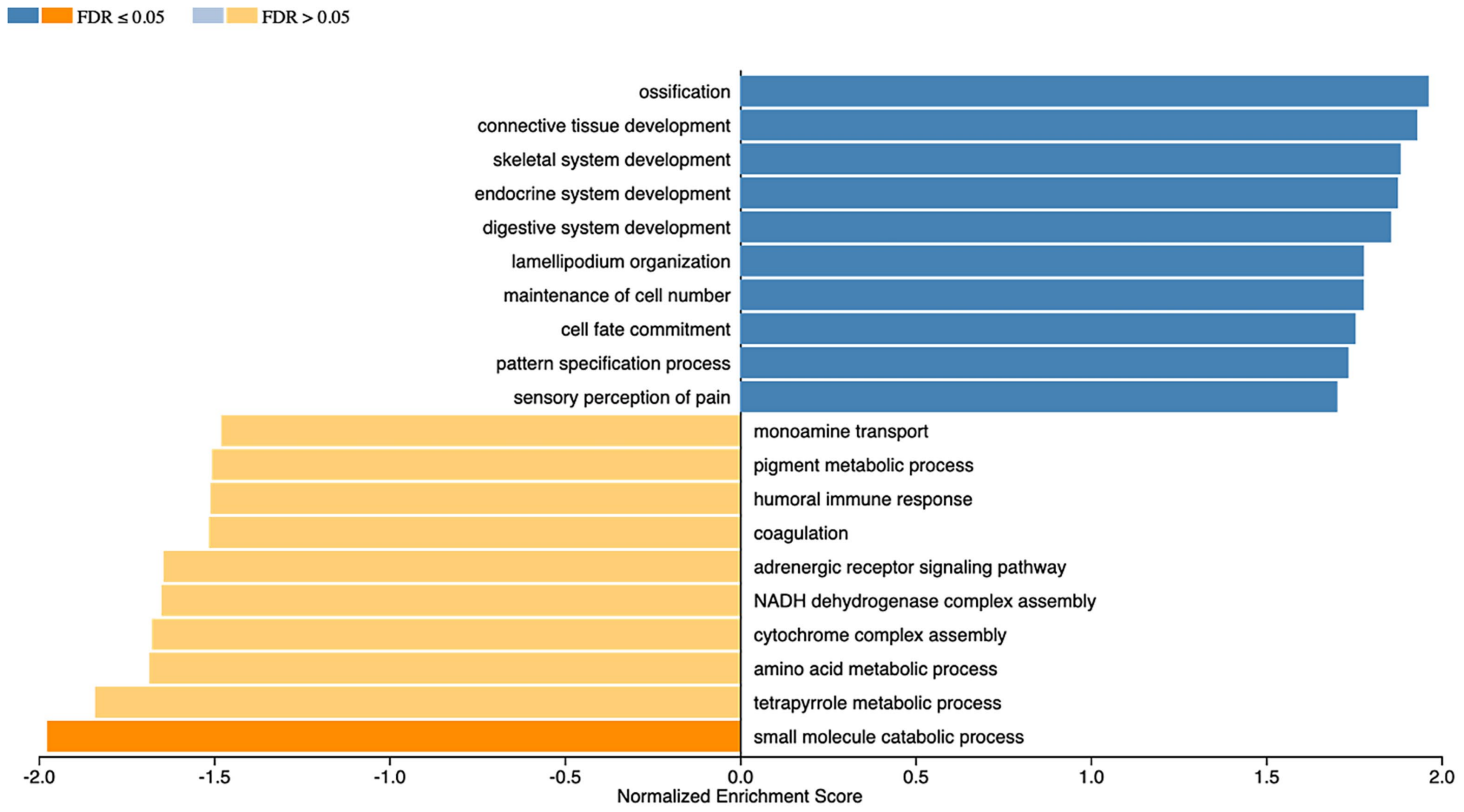
Gene set enrichment analysis-based pathway on biological processes GO terms of expressed genes in the liver of beef calves. Liver biopsies were performed on d 56 on calves randomly selected on day 0 for transcriptomics analysis [calves born from cows without access to shade (NS, *n* = 1*0; steers = 5 an*d heifers = 5) or given access to shade (SS, *n* = 10; steers = 5 and heifers = 5)]. The normalized enrichment score (NES) of top enriched (blue bars) and top depleted (orange bars) pathways are based on the comparison of NS vs. SS calves (FDR ≤ 0.05).

GO Biological Process (Figure 6) analysis demonstrated that NS calves exhibited enrichment in several functional categories related to growth, development, and cellular organization, including ossification, connective tissue development, skeletal system development, and endocrine system development. Conversely, depleted processes (negative NES) in NS calves consisted of small molecule catabolic process (FDR < 0.05). Although over-represented, immune-related processes, including humoral immune responses were above the significance level (FDR = 0.13).

**Figure 7:**
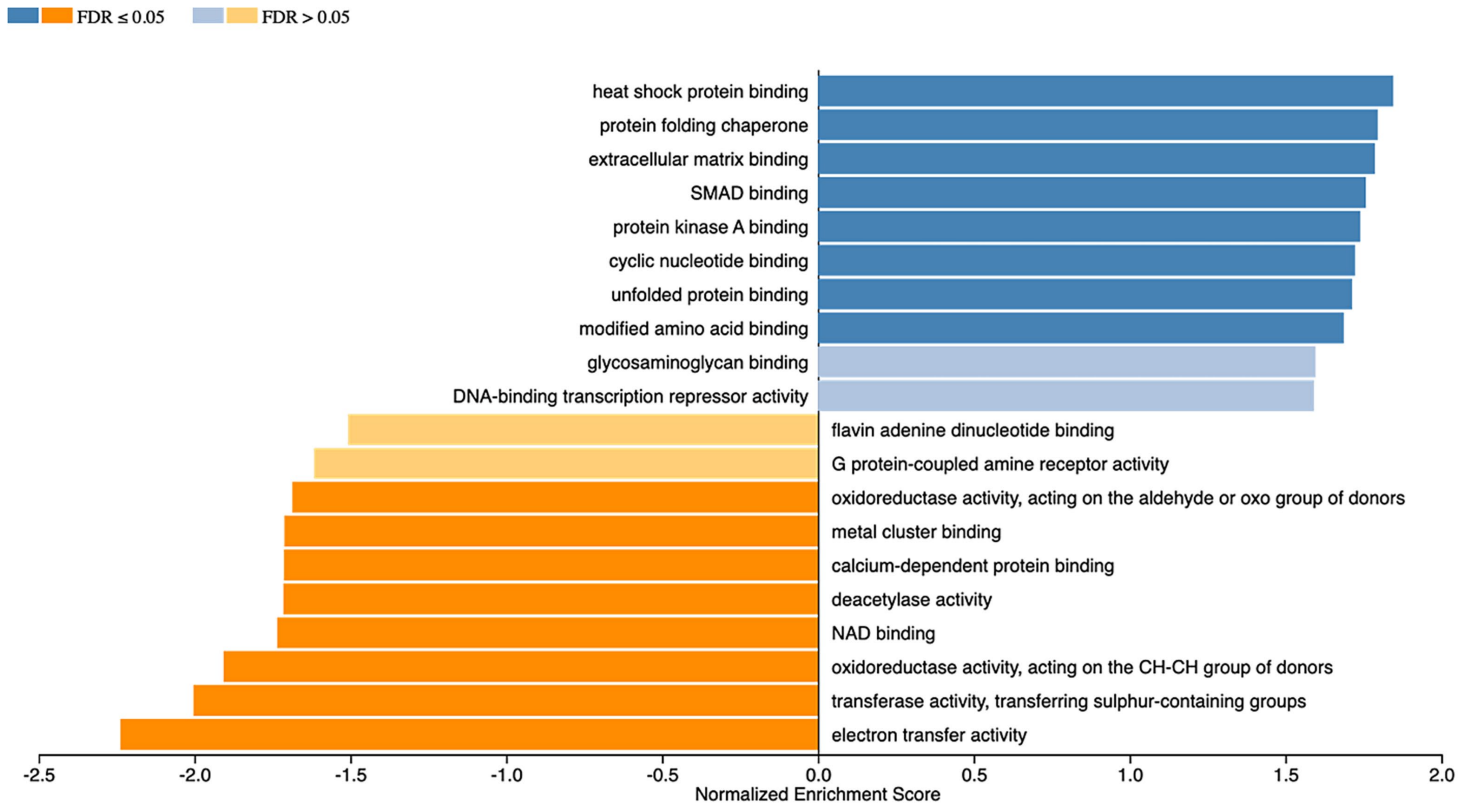
Gene set enrichment analysis-based pathway molecular function GO of expressed genes in the liver of beef calves. Liver biopsies were performed on d 56 on calves randomly selected on day 0 for transcriptomics analysis [calves born from cows without access to shade (NS, *n* = 10; steers = 5 and heifers = 5) or given access to shade (SS, *n* = 10; steers = 5 and heifers = 5)]. The normalized enrichment score (NES) of top enriched (blue bars) and top depleted (orange bars) pathways are based on the comparison of NS vs. SS calves (FDR ≤ 0.05).

GO Molecular Function analysis revealed several terms over-represented in the NS calves, including those related to heat shock protein binding, protein folding chaperone activity, and extracellular matrix binding (Figure 7). In contrast, depleted functions in NS calves involved molecular functions primarily related to oxidoreductase activity, including multiple oxidoreductase subclasses acting on aldehyde or CH-OH groups, as well as electron transfer activity, NAD binding, metal cluster binding, calcium-dependent protein binding, deacetylase activity, and various catalytic activities.

## 4 Discussion

Research has extensively documented the short- and long-term effects of heat stress in cattle, with most work conducted in dairy systems (3, 10, 25). In the present study, treatment differences did not compare offspring born to heat-stressed vs. thermoneutral cows; instead, calves born to cows exposed to different levels of heat load by removing heat abatement during gestation (i.e., shade vs. no shade). In a previous study, we observed reduced performance in cows managed with no access to shade during gestation, including decreased BW and BCS, as well as disruptions in metabolic profile (4). Short-term consequences for neonatal calves born to no-shade cows included lower birth weight and reduced IgG concentrations compared to calves born to shaded cows (4). In this follow up paper, we assessed the long-term consequences during weaning and transition to the backgrounding phase of calves exposed to different *in utero* heat load. This phase is particularly critical because summer-weaned calves must cope simultaneously with heat stress and the substantial stress of weaning, which is widely recognized as one of the most challenging periods in the beef cattle cycle (11).

In this study, SS calves were heavier at weaning and remained heavier through the end of the study, with no differences in overall ADG between groups. This demonstrates that NS calves did not fully compensate for their initial weight disadvantage. Similarly to this study, Halli et al. (26) reported that calves born to cows (a dual-purpose breed) exposed to severe heat stress during late gestation were lighter at 365 days of age compared with calves born to cows exposed to moderate heat stress. Evidence from dairy systems further supports the long-term growth disadvantages associated with prenatal heat stress, as calves exposed in utero often exhibit persistent reductions in BW well beyond the neonatal period. For instance, Dado-Senn et al. (6) found lower post-weaning BW in heifers born to heat-stressed dams and Monteiro et al. (27) reported that heifers born to heat-stressed cows remained lighter up to 1 year of age, although their total weight gain over time was similar to that of heifers born to cooled cows. Conversely, Izquierdo et al. (8) working with early-weaned beef calves reported that calves born to shaded cows were lighter post-weaning and tended to have lower ADG than calves born to NS cows.

We identified a limited number of DEGs, which may be due to the interval between the maternal insult and the assessment of gene expression in the offspring. When adjusting for sex, only two genes were differentially expressed between treatments, whereas sex-stratified analyses identified nine DEGs in females and none in males, suggesting a sex-dependent transcriptional response. The greater number of DEGs in females may reflect a more coordinated response, potentially driven by differences in endocrine or metabolic regulation between sexes. The limited number of DEGs in the sex-adjusted model likely reflects reduced power when accounting for sex as a covariate, particularly if gene expression changes are sex-specific and thus diluted in the combined analysis. Similarly, the absence of DEGs in males may indicate a more stable transcriptional response or may be due to limited power to detect subtle changes. Given the relatively small number of DEGs overall, we focused our discussion on gene set enrichment analysis (GSEA), which captures coordinated changes across gene sets and provides greater sensitivity to detect biologically meaningful patterns.

Based on the GSEA, results from KEGG analysis showed that NS calves exhibited an upregulation of genes underlying pathways related to skeletal, bone, endocrine, and digestive system development. This is likely due to these calves being on a different growth trajectory, so such pathway activation is typical of animals positioned for compensatory growth (28, 29). Nevertheless, despite these molecular indicators, NS calves did not overcome their initial BW deficit, as reflected by similar ADG between groups. During the first 3 days following weaning, however, NS calves tended to lose more weight than SS calves, which coincided with lower SUN concentrations in NS calves from d 0 to d 7. Serum urea N reflects the balance between nitrogen intake and utilization, increasing with greater dietary protein supply or enhanced amino acid catabolism during tissue mobilization for gluconeogenesis, for example (30). In the early post-weaning period, when feed intake is often depressed, reductions in SUN are associated with decreased nitrogen intake rather than muscle breakdown. Accordingly, the lower SUN values in NS calves are likely due to reduced feed intake, suggesting that differences in early weight change were driven largely by gut fill. Importantly, this transient post-weaning decline in performance did not alter the overall ADG trajectory across the background phase.

NS calves exhibited greater BVDV-1 titers and seroconversion than SS calves, with no differences in stress markers. Izquierdo et al. (8) partially corroborate these findings: they reported no differences in cortisol, seroconversion, or serum titers for BVDV-1, IBR, or PI-3, but NS calves tended to have greater BRSV titers and seroconversion. Interpretation of antibody responses in the present study should also consider the vaccination protocol employed, as calves received a single vaccination approximately 21 days before weaning, with no booster administered thereafter. This may have limited antigenic stimulation and resulted in a generally mild antibody response, as serum titers were overall lower than values commonly reported in calves weaned under comparable conditions (31). Research examining the effects of maternal heat stress on offspring immunity has largely been conducted in dairy cattle, with a predominant focus on passive immune transfer, particularly colostral IgG concentration and absorption (32–34). Consequently, studies directly assessing active immune competence in beef calves exposed to in-utero heat stress remain scarce, as well as the underlying biological mechanisms. Further research is therefore warranted to elucidate the direct impacts of in-utero heat stress on long-term immune competence, as current evidence remains inconclusive.

Another objective of this study was to determine whether NS and SS calves differed in thermotolerance when exposed to high THI during the summer. From d 77 to d 84, NS heifers exhibited greater average IT than SS calves, although no differences were detected in respiration rate. Importantly, although significant, the magnitude of this difference (|0.3| ± 0.04 °C) was not biologically sufficient to impair ADG. To our knowledge, this is the first study to explore thermotolerance mechanisms in beef calves with distinct in-utero heat stress exposures. All calves were managed as a single group to ensure equal access to heat abatement, which consisted of abundant natural shade. Thus, differences in thermotolerance observed herein are likely attributed to in-utero management rather than postnatal environmental variation. Supporting these observations, Dado-Senn et al. (10) reported higher rectal temperatures and respiration rates in dairy calves born to heat-stressed cows, and Laporta et al. (34) found greater rectal temperature at birth and at 28 days in calves born to heat-stressed dams compared with cooled dams. Notably, the calves in those studies were much younger than those in the present study, so differences may reflect additional physiological factors beyond thermotolerance alone. Similar responses have been reported in other species. Conversely, Ahmed et al. (9) observed that dairy cows born to heat-stressed dams tended to have lower late-afternoon rectal temperatures, potentially indicating a thermotolerant mechanism, although this difference was limited to a single time point and therefore represents weak evidence. Inconsistencies can be attributed to different ages, heat stress exposure, or physiological state, highlighting the need for more research on the impacts of in-utero heat stress on offspring thermotolerance, specifically in beef cattle. In the present study, body temperature was measured only in the heifers (via intravaginal temperature), which is a limitation, although sex was equally distributed across groups. Additionally, behavioral responses may affect thermotolerance responses (35), and it was not assessed in this experiment. Despite equal access to shade, calves may have differed in grazing and idling patterns, thereby affecting individual heat load. Future studies integrating physiological and behavioral measures will be essential to determine whether observed thermotolerance differences are due solely to physiological mechanisms, solely to behavioral strategies, or to a combination of both.

Molecular function GO analysis revealed that calves from NS cows showed enrichment in several terms associated with heat-shock-protein binding, which are considered key determinants of thermotolerance in livestock (36). Heat shock proteins play a central role in cellular protection against heat stress by acting as molecular chaperones that refold denatured proteins, prevent aggregation of damaged proteins, and help maintain protein homeostasis under thermal stress (37). In addition, NS calves showed enrichment of the thyroid hormone signaling pathway (although FDR not significant), which is central to metabolic regulation and heat production and is known to influence thermoregulatory capacity (38). Consistent with these molecular signatures, functional over-representation analysis of DEGs showed greater activation and regulation of vasoconstriction pathways in NS heifers. When interpreted alongside the higher intravaginal temperatures observed in NS calves, these results collectively suggest that this group experienced greater difficulty maintaining thermal homeostasis, likely resulting in compensatory activation of key thermoregulatory and metabolic pathways.

These findings align with broader evidence indicating that maternal heat stress induces long-lasting transcriptomic and epigenetic reprogramming in dairy offspring (39). Notably, several of the biological processes identified as differentially enriched in our study overlap with pathways previously associated with intrinsic thermotolerance. Freitas et al. (40) observed enrichment of vasoconstriction regulation, hemostasis, and clotting cascade pathways in thermotolerant Caracu cattle compared to less thermotolerant counterparts, suggesting that efficient vascular regulation and coagulation balance are key components of adaptive heat tolerance. In contrast, NS calves in the present study exhibited downregulation of multiple coagulation-related genes (e.g., *FGA, FGB, FGG*) and enrichment of vascular and hemostatic biological processes, particularly in females. Importantly, NS heifers exhibited greater intravaginal temperature, indicating impaired thermal homeostasis compared to SS. This finding provides critical context for the observed transcriptional patterns. Therefore, in this study functional over-representation of biological processes related to vasoconstriction and coagulation, together with enrichment of heat shock protein binding and chaperone-related molecular functions, likely reflects sustained cellular and vascular stress rather than enhanced thermoregulatory capacity. Additionally, key molecular functions related to mitochondrial redox balance and catalytic activity were depleted, which is attributed to reduced metabolic flexibility and impaired capacity to dissipate endogenous heat. Importantly, in this case, enrichment of heat shock protein binding indicates increased cellular reliance on chaperone-mediated protein maintenance rather than increased heat shock protein abundance per se, as observed in proteomic analyses of high thermotolerant cattle (40). Similar hepatic transcriptional responses have been reported in heat-stressed dairy cows, in which suppression of oxidative phosphorylation pathways occurred alongside activation of protein folding and chaperone-related processes, reflecting metabolic dysfunction rather than thermotolerance (41). Taken together, the elevated temperature and transcriptomic profile of NS heifers support the interpretation that prenatal heat stress induces long-term physiological constraints on thermoregulatory mechanisms pathways, rather than programming a thermotolerant phenotype.

## 5 Conclusion

Removing maternal heat abatement during gestation led to long-term growth disadvantages in offspring, as calves born to no-shade cows were lighter at weaning and remained lighter through the backgrounding phase, despite no differences in overall ADG between groups. Gene enrichment and functional over-representation analyses revealed alterations in metabolic and growth-related pathways in NS calves, including enrichment of heat-shock protein binding, protein folding chaperone, and greater activation and regulation of vasoconstriction pathways. These molecular changes, together with elevated intravaginal temperature in NS compared to SS heifers, suggest that *in utero* heat stress altered thermoregulatory control. Calves exposed to greater prenatal heat load also exhibited differences in humoral immune responses, as indicated by greater BVDV-1 titers and seroconversion. Further studies are warranted to deeper investigate the relationship between maternal heat stress and offspring immunity responses later in life. Collectively, growth, physiological, and transcriptomic findings support the concept that prenatal heat stress induces lasting modifications in growth, thermoregulatory, and metabolic pathways, that may ultimately influence the ability of beef calves to cope with challenges later in life. These results highlight the importance of maternal heat abatement management during gestation and provide mechanistic insight into how gestational heat stress may shape offspring postnatal growth and resilience in grazing systems.

## Data Availability

All relevant data are within the paper and its Supplementary Information files. All RNA-sequencing data is publicly available on NCBI’s Gene Expression Omnibus through GEO Series accession number GSE318541.
