# Supplementary material for: Maternal heat abatement during gestation alters growth, immunity, thermotolerance, and hepatic gene expression in beef offspring during backgrounding
Source: Front Vet Sci. 2026 May 29;13:1849652. doi: 10.3389/fvets.2026.1849652 (PMC13259762; doi:10.3389/fvets.2026.1849652)
Supplement: Supplementary file 1 [file Data_Sheet_1.DOCX]

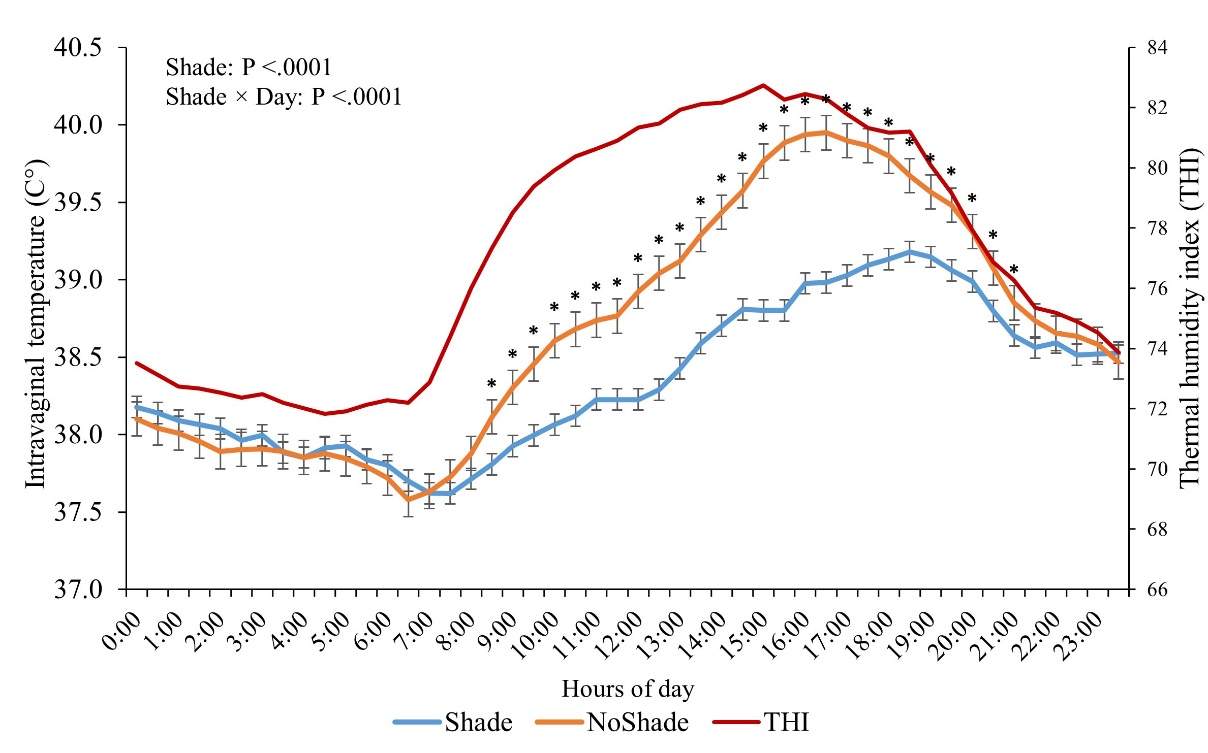


Supplementary Image 1

Intravaginal temperature (°C) of Angus-cross cows with or without access to shade from days 0 to 180 (150 pre-partum until 30 days postpartum) from d 53 to 60 (Panel A) and d 83 to 90 (Panel B). Intravaginal temperature was averaged at 30-min intervals and then across days from 54 to 59 before statistical analysis. Interaction between Shade × Hour was detected from d 55 to 59 (P < 0.001). Average intravaginal temperature differed between cows with access to shade and those without, averaging 38.3 vs. 38.7 ± 0.07 °C, respectively. During the hottest hours of the day (1400 to 1800 h), intravaginal temperature was also lower in shaded compared with non-shaded cows (39.0 vs. 39.9 ± 0.08 °C)


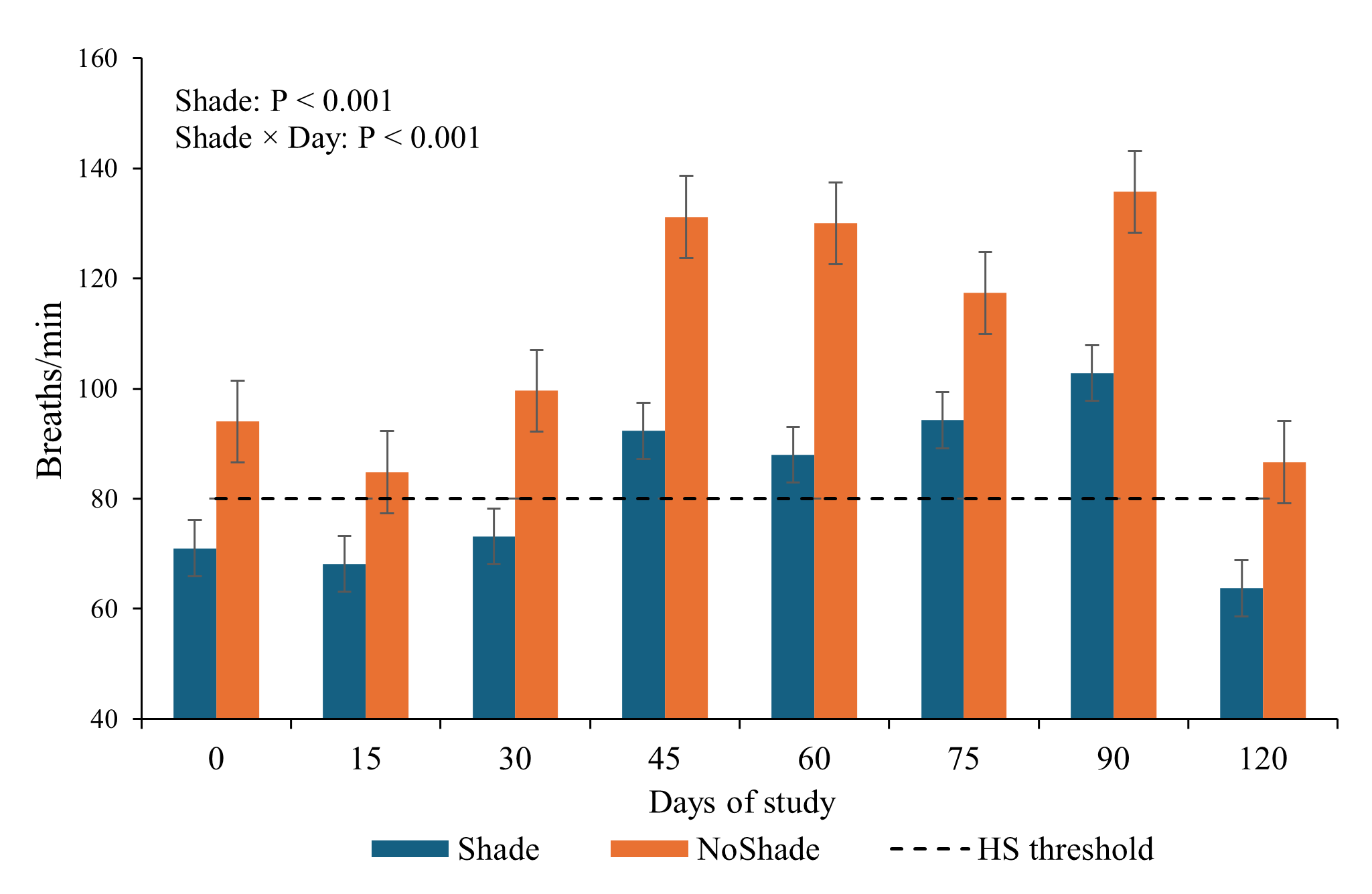


Supplementary Image 2

Respiration rate of Angus-cross cows with or without access to shade from days 0 to 180 (150 pre-partum until 30 days postpartum) every 14-days. Respiration rate was measured by visually counting flank movements of each cow for 1 min. Heat stress (HS) threshold (RR ≥ 80 bpm). Average respiration rate differed between cows with access to shade and those without, averaging 82 vs. 110 ± 5.7 breaths/min.
